# Cost analysis comparing guideline-oriented biopsychosocial management to usual care for low-back pain: a cluster-randomized trial in occupational health primary care

**DOI:** 10.5271/sjweh.4212

**Published:** 2025-05-01

**Authors:** Maija Paukkunen, Jaro Karppinen, Birgitta Öberg, Leena Ala-Mursula, Eveliina Heikkala, Katja Ryynänen, Riikka Holopainen, Samuel Booth, Neill Booth, Allan Abbott

**Affiliations:** 1 Department of Health, Medicine and Caring Sciences, Linköping University, Linköping, Sweden.; 2 Research Unit of Health Sciences and Technology, University of Oulu, Oulu, Finland.; 3 Wellbeing Services County of South Karelia, Lappeenranta, Finland.; 4 Research Unit of Population Health, University of Oulu, Oulu, Finland.; 5 Wellbeing Services County of Lapland, Rovaniemi, Finland.; 6 University of Jyväskylä, Jyväskylä, Finland.; 7 Wellbeing Services County Southern Savo, Mikkeli, Finland.; 8 Faculty of Social Sciences, Tampere University, Tampere, Finland.; 9 Department of Orthopaedics, Linköping University Hospital, Linköping, Sweden.

**Keywords:** health services research, implementation research, occupational health service, pain, resource, return to work, risk stratification, screening, workability, Örebro Musculoskeletal Pain Screening Questionnaire

## Abstract

**Objectives:**

This study aimed to investigate the effect of a brief training intervention for occupational health services (OHS) professionals on multiprofessional resource utilization and the costs of biopsychosocial management of patients with low-back pain (LBP) compared to usual care among all participants and those in work disability-based risk groups.

**Methods:**

OHS utilization and back-related sick leave data were collected from electronic patient records over one-year follow-up comparing 232 patients in the intervention arm and 80 control-arm patients, stratified for risk of work disability based on the Örebro Musculoskeletal Pain Screening Questionnaire. We estimated costs using linear mixed models by multiplying unit costs (in euros) by each type of OHS resource use (visits to physicians, physiotherapists, nurses, use of imaging) and the number of sick leaves. Estimated mean cost differences with confidence intervals (CI) were reported using bootstrapping to deal with skewed cost data.

**Results:**

The median number of visits to physicians and physiotherapists in the intervention versus control arms was 1 [interquartile range (IQR) 0–3] and 2 (IQR 1–4) versus 2 (IQR 1–3) and 1 (IQR 0–2), respectively. The intervention arm accrued lower physician costs (€-43, 95% CI €-82– -3, P=0.034) and higher physiotherapist costs (€55, 95% CI €26–84, P<0.001) compared to the control arm. There was no statistically significant difference in average total costs between the arms (€-1908, 95% CI €-6734–2919). In the low- and medium-risk groups of work disability, physiotherapist costs were higher in the intervention than control arm, but no statistically significant differences were observed between the arms in the total resource utilization or sickness absence costs.

**Conclusions:**

Brief biopsychosocial training may support shifting OHS resources towards multiprofessional physiotherapist-driven care, instead of solely physician-driven care, for management of patients with LBP in differing risk groups of work disability with no substantial differences in total costs.

Low-back pain (LBP) is one of the most common work-related conditions and can be quite costly (1–4). Therefore, identifying a mix of services that are both effective and cost-effective is of great importance in modern occupational healthcare. Most patients with new episodes of LBP recover quickly, however recurrence is common and, for a small proportion, LBP becomes persistent and disabling (5). Long periods of sickness absence predict disability pensions, but even relatively short sickness absences can be predictive of disability (6). In a registry-based real-world study, the prevalence of chronic LBP in 2011–2017 was 2.7% in specialized care settings and 5.8% in combined specialized and primary care in Finland. Importantly, the specialized care patients had lower socioeconomic status, employment rates and disposable income levels and higher likelihood of comorbidities compared to their matched population-based controls (7). These findings highlight the need for more comprehensive strategies to mitigate disability from LBP.

In recent years, multiple tools have been developed for the prediction of work disability risk among patients with musculoskeletal pain. One of these is the validated Örebro Musculoskeletal Pain Screening Questionnaire (ÖMPSQ) (8), which was found to have "acceptable" performance for prediction of disability outcomes but "excellent" performance concerning absenteeism (9). A 10-item short form of the ÖMPSQ (ÖMPSQ-SF) extracted from the 25-item full version (10) was found to be "good" in predicting work disability (11). In a population-based cohort, the high-risk group according to ÖMPSQ-SF had 7.5-times higher number of sick leave days and 16.1-times higher odds of disability pension over a 2-year follow-up compared to the low-risk subgroup (11). Importantly, a stratified approach, with more focus on high-risk patients, has been found to be effective in reducing long-term disability among patients with musculoskeletal pain (12).

The occupational health services (OHS) provide an excellent context for developing multiprofessional collaboration by being oriented towards workability support, well connected to workplaces, and in close partnership with different stakeholders. Under the Finnish Occupational Healthcare Act, all employers must provide OHS to their employees to foster safety and health and prevent work disability. In addition, most employers voluntarily purchase primary healthcare services and sometimes specialist consultations from the OHS, as agreed in the varying contracts between the OHS providers and the employers. In 2021, 90.1% of the Finland’s employed workforce (1.98 million people) were covered by preventive OHS and further 90% of those had also access to occupational health (OH) primary care services (13).

While all major guidelines on the management of LBP recommend a biopsychosocial (BPS) management approach (14), the training for OH professionals to use this approach has not been widely available. Previously, we have reported the clinical effectiveness of a brief training intervention for OH professionals in implementation of guideline-oriented and risk stratified BPS management of LBP in a cluster-randomized study with 1-year follow-up (15, 16). In the trial, we observed no clinically relevant differences between the patient-reported outcome measures of the patients recruited by the healthcare practitioner trained in BPS management of LBP and those of the patients recruited through usual OHS over one-year follow-up (15). In the present study, we have proceeded to conduct an economic evaluation (17) of the aforementioned trial (15). Specifically, we have investigated the effects on multiprofessional resource utilization and associated costs to employers (i) among all LBP patients and (ii) in patient groups stratified for work disability risk according to ÖMPSQ-SF.

## Methods

### Study setting and participants

The OHS are important providers of primary care in Finland, delivering services of OH physicians, nurses, physiotherapists, and psychologists. At the time of this study, the latter two were available according to OH physicians’ and nurses’ referrals, but, since 2022, OH physiotherapists have also been first-contact clinicians.

As detailed previously (15), a total of 27 OHS units from six different private and publicly funded companies participated in the study. For each participating company, the units were randomized to either an intervention or to a control arm. BPS training for OH physicians and physiotherapists lasted 3–7 days, depending on the professional. A total of 28 professionals attended the initial training in 2017, 21 in 2018, and 17 at both times, while 32 professionals attended at least one of the trainings. The brief training (4 days in the initial training in September 2017 and 3 days in the booster training in June 2018, in total 44 hours) intervention for OH professionals in implementing guideline-oriented stratified BPS management was held by world-leading experts in LBP management (16). The extensiveness of the training was similar to conventional continuing education workshops for professionals and feasible to accomplish. In the supplementary material (www.sjweh.fi/article/4212), figure S1 presents the infographic of guideline-based BPS patient management of LBP for which the professionals in the intervention arm received training. The training included presentations, discussion, and practical training with patient demonstrations about clinical assessment, clinical reasoning, therapeutic alliance, pain behaviors, fear-avoidance, imaging, communication skills and key management principles (16). Professionals working in the control arm were not exposed to the BPS training and were therefore expected to treat patients as usual.

The professionals in both the intervention and control arms recruited the patients for the study from 25 September 2017 to 1 December 2018. We included all patients aged 18–65 with LBP with or without radicular pain. Exclusion criteria were suspicion of a serious underlying cause of LBP and need for urgent care (15).

**Figure 1:**
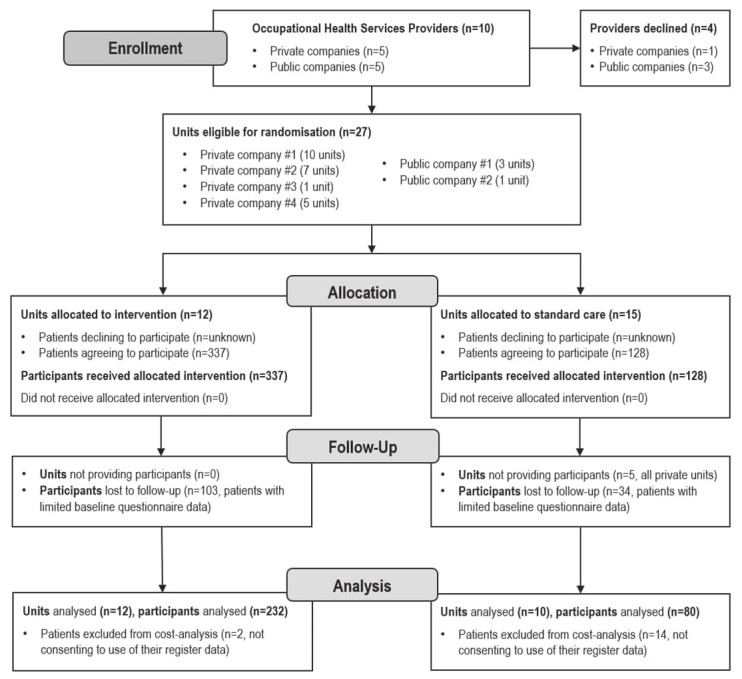
Flow chart of the phases of the cluster randomized controlled trial used in the cost analysis.

Analyses are based on the data of 312 patients (232 in the intervention and 80 in the control arm) who responded to the electronic questionnaire and provided written informed consent to participate and for their register data to be accessed. The intervention arm included 12 OHS units. Of the original 15 control units, patients were recruited from 10 OHS units. Figure 1 presents the CONSORT flow diagram for the cluster-randomized controlled trial.

The ethics committee of the Northern Ostrobothnia Hospital District has approved the research (79/2017), which was conducted according to the Declaration of Helsinki and retrospectively registered in the BioMed Central register (ISRCTN11875357). The only deviation from the protocol emerged from denied access to national register data on work disabilities due to changes in data regulations beyond our control. The University of Oulu was the registrar of the study (16).

### OHS visits, imaging, and back-related sick leave days

Patient-level data on OH primary care utilization was extracted from electronic patient records (EPR). Data were collected retrospectively from individual EPR one year after the date of consent obtaining the number of face-to-face visits and remote visits to OH professionals (physicians, physiotherapists and nurses) and the associated ICD-10 M40-M54 diagnoses. Physicians included the specialist OH physicians and physicians without specialization in OH but working in OH primary care. We excluded all general medical examinations, mandatory occupational safety examinations, telephone calls for booking appointments or prescription renewals and group sessions.

Multiprofessional physiotherapist-driven care was coded as a variable, based on records, to show at least one physiotherapist visit in addition to at least one visit to a physician or a nurse in OHS. The number of imaging examinations and number of sick leave days accrued according to ICD10 M40-M54 diagnoses within the 12-month follow-up were extracted from the EPR.

### Costs

Limited societal and healthcare perspectives were applied in our analysis. Societal perspectives typically include estimated effects on productivity and, in this study, sick leaves were used as an indicator of productivity costs, supplementing our healthcare perspective on the use of OHS resources. The chosen healthcare perspective is that of an OHS payer since employers paid the costs of the use of OHS over the trial follow-up. Employers can later apply for partial (50–60%) reimbursements from KELA, the social insurance institution. In Finland, employers pay all costs for the 1–10 first sick leave days, thereafter they receive partial reimbursement if they continue paying wages during later part- or full-time sick leave.

We estimated OHS costs by multiplying healthcare unit costs (in euros) (18) by each type of OHS resource use (supplementary table S1). In 2017, the Confederation of Finnish Industries estimated the cost of a sick leave day to be €350. We summarized the costs to the following categories and used them as study outcomes: visits to physicians, physiotherapists and nurses; medical imaging or other diagnostic examinations [including radiograph (X-ray)/magnetic resonance imaging (MRI)/electroneurography (ENG)/electromyoneurography (ENMG)]; total OHS resource use; and sick leave days due to LBP.

Total costs, which included all these costs, were also calculated. All cost variables were treated as continuous.

In our trial, the OHS service providers did not pay for the BPS training intervention and participants could attend without fees during their normal working hours. In additional sensitivity analysis, we estimated the direct cost of the initial training to be €2500 and the direct cost of the booster training to be €1000, based on the trainers’ salaries for the time used. Unfortunately, we were only able to estimate these associated indirect productivity costs for employers because we did not have access to data on the participants’ salaries or other potential expenses, such as travel costs. A one-time investment of €29 222 for delivering the training was included in the additional analysis, equivalent to €126 per patient recruited from the intervention arm. This calculation also includes estimates of professionals’ salaries for the time spent attending the training.

### Work disability risk according to the Örebro Musculoskeletal Pain Screening Questionnaire - Short Form

We used the 10-item ÖMPSQ-SF (supplementary figure S2) (10), which has been translated and validated in Finnish (19) as a risk stratification tool for work disability. The items of ÖMPSQ-SF include: (i) the duration of pain(s), (ii) pain rating, (iii) the ability to do light work, (iv) the ability to sleep at night, (v) feelings of anxiety, (vi) feelings of depression, (vii) the perceived risk of pain becoming chronic, (viii) perceived opportunities to return to work and (ix and x) fear-avoidance beliefs. Each item is scored 0–10, and respondents are divided into three groups according to their total scores: (i) low (0–39 points); (ii) medium (40–49 points); and (iii) high risk (50–100 points) (10, 20).

### Baseline characteristics

Descriptive characteristics in relation to demographics, general health, LBP and workability were surveyed at baseline (15) including age, gender, body mass index (BMI, self-reported weight per squared height kg/m^2^), smoking, self-rated health (EuroQol (EQ)-5D, 0–100 visual analogue scale), health-related quality of life using the EQ-5D-3L, the Depression Scale (DEPS), pain duration, the Oswestry Disability Index (ODI), workability (0–10 numerical rating scale), number of sick leave days due to LBP during preceding three months and fear of physical activity or work (Fear-Avoidance Beliefs Questionnaire, FABQ).

### Statistical methods

The distribution of included variables was described using means with standard deviations (SD) for normally distributed continuous/count variables, medians with interquartile ranges (IQR) for non-normally distributed continuous/count variables and using frequencies (N) with percentages (%) for categorical variables. The statistical significance of the differences between intervention and control groups was estimated using the independent-samples T test, Chi-square and Mann-Whitney U tests, as appropriate, in the total study sample and within the ÖMPSQ-SF risk groups. As a supplementary analysis, we compared costs between ÖMPSQ-SF risk groups in the total sample and estimated the statistical significance of potential differences using Kruskal-Wallis test. P-values <0.05 were considered statistically significant.

Two-level linear mixed models, with fixed effects for group (intervention versus control) and random effects for unit, were used to analyze the associations between trial arms and costs among all LBP patients and within ÖMPSQ-SF risk groups. Mean differences in the costs between the trial arms were reported with accompanying confidence intervals (CI) and associated P-values. Bootstrapping with 2500 replications was used to attempt to deal with skewness in the distribution of costs. Analyses included unadjusted and adjusted models, and confounders for each model were selected from baseline variables based on a statistically significant difference in the distribution between trial arms and were as follows: duration of pain and pain-related fear for physical activity for all patients, age for ÖMPSQ-SF low-risk group, general health for medium-risk group, and duration of pain for high-risk group. Statistical analyses were conducted using SPSS Statistics version 29 (IBM Corp, Armonk, NY, USA).

## Results

Patient characteristics (total N=312) are shown in table 1a. In all, 58% were women and the age range was 21–64 years. A total of 52.9% of the participants belonged to the ÖMPSQ-SF low-risk, 21.5% to the medium-risk and 25.6% to the high-risk group. Statistically significant differences between the trial arms in the ÖMPSQ-SF groups at baseline are shown in tables 1b–d.

**Table 1a:** Baseline characteristics of participants in the trial arms. **Bolded scores denote statistical significance with P<0.05**. [EQ=EuroQol score; FABQ=Fear Avoidance Beliefs Questionnaire; IQR=interquartile range; ODI=Oswestry Disability Index; SD=standard deviation].

			Total (N=312)							
			Intervention (N=232)				Control (N=80)			P-value
			% (N) ^a^	Mean (SD) ^b^	Median (IQR) ^c^		% (N) ^a^	Mean (SD) ^b^	Median (IQR) ^c^	
General health										
	Age (years)			44.6 (9.4)				45.9 (11.2)		0.341
	Female sex		54.7 (128)				65.4 (53)			0.092
	Body mass index (kg/m^2^)			27.7 (5.3)				27.3 (4.3)		0.527
	Smoking		15.0 (35)				16.0 (13)			0.814
	Self-rated health (1–100)				75 (65–85)				80 (65–85)	0.327
	EQ-5D (0–1)				0.76 (0.69–0.80)				0.76 (0.69–0.80)	0.566
	Depression Scale				4 (2–9)				4 (2–9)	0.849
Low-back pain-related										
	Duration of pain									
		<2 weeks	**11.1 (26)**				**19.8 (16)**			**0.017**
		2–12 weeks	**32.9 (77)**				**37.0 (30)**			
		>3–12 months	**21.4 (50)**				**25.9 (21)**			
		>12 months	**34.6 (81)**				**17.3 (14)**			
	ODI (0–100)				20 (12–28)				20 (12–29)	0.877
Workability										
	Workability (0–10)				8 (6—9)				8 (6–8)	0.542
	Sick leave days ^d^			49.1 (115)				59.3 (48)		0.116
	Sick leave days ^d^				8 (4–29)				14 (4–23)	0.572
	FABQ - work				11 (4–19)				13 (5–24)	0.097
	FABQ - physical activity				**12 (8–14)**				**14 (9–17)**	**0.012**

^a^ P-value for between-group difference from Chi square test. ^b^ P-value for between-group difference from independent-samples T test. ^c^ P-value for between-group difference from Mann-Whitney U test. ^d^ Number of low-back pain related sick leave days during preceding 3 months to low-back pain during preceding 3 months.

**Table 1b:** Baseline characteristics of participants in the trial arms stratified by low-risk work disability groups based on the short form of Örebro Musculoskeletal Pain Screening Questionnaire (ÖMPSQ-SF). **Bolded scores denote statistical significance with P<0.05**. [EQ=EuroQol score; FABQ=Fear Avoidance Beliefs Questionnaire; IQR=interquartile range; ODI=Oswestry Disability Index; SD=standard deviation].

			Low-risk (N=165)											
			Intervention (N=127)						Control (N=38)					P-value
			% (N) ^a^		Mean (SD) ^b^		Median (IQR) ^c^		% (N) ^a^		Mean (SD) ^b^		Median (IQR) ^c^	
General health														
	Age (years)				**43.7 (9.4)**						**45.0 (12.5)**			**<0.001**
	Female sex		55.9 (71)						60.5 (23)					0.614
	Body mass index (kg/m^2^)				26.9 (5.1)						26.8 (4.3)			0.742
	Smoking		14.2 (18)						15.8 (6)					0.804
	Self-rated health (1–100)						80 (75–90)						85 (79–90)	0.513
	EQ-5D (0–1)						0.86 (0.80–0.86)						0.86 (0.80–0.86)	0.469
	Depression Scale						3 (1–4)						3 (1–5)	0.942
Low-back pain-related														
	Duration of pain													
		<2 weeks	18.1 (23)						31.6 (12)					0.115
		2–12 weeks	40.2 (51)						36.8 (14)					
		>3–12 months	19.7 (25)						23.7 (9)					
		>12 months	22 (28)						7.9 (3)					
	ODI (0–100)						12 (8–18)						12 (6–16)	0.313
Workability														
	Workability (0–10)						8 (8–9)						8 (7.8–9)	0.173
	Sick leave days ^d^		44.9 (57)						47.4 (18)					0.787
	Sick leave ^d^						7 (3–14)						10.5 (3–25.5)	0.482
	FABQ - work						8 (2–14)						10.5 (1–21)	0.181
	FABQ - physical activity						9 (6–13)						11 (5.5–16)	0.208

^a^ P-value for between-group difference from Chi square test. ^b^ P-value for between-group difference from independent-samples T test. ^c^ P-value for between-group difference from Mann-Whitney U test. ^d^ Number of low-back pain related sick leave days during preceding 3 months.

**Table 1c:** Baseline characteristics of participants in the trial arms stratified by medium-risk work disability groups based on the short form of Örebro Musculoskeletal Pain Screening Questionnaire (ÖMPSQ-SF). **Bolded scores denote statistical significance with P<0.05**. [EQ=EuroQol score; FABQ=Fear Avoidance Beliefs Questionnaire; IQR=interquartile range; ODI=Oswestry Disability Index; SD=standard deviation].

			Medium-risk (N=67)											
			Intervention (N=47)						Control (N=20)					P-value
			% (N) ^a^		Mean (SD) ^b^		Median (IQR) ^c^		% (N) ^a^		Mean (SD) ^b^		Median (IQR) ^c^	
General health														
	Age (years)				44.1 (10.1)						48.8 (9.1)			0.378
	Female sex		48.9 (23)						70 (14)					0.113
	Body mass index (kg/m^2^)				28.6 (4.9)						27.4 (4.7)			0.609
	Smoking		14.9 (7)						10 (2)					0.591
	Self-rated health (1–100)						**70 (60–80)**						**80 (71–85)**	**0.020**
	EQ-5D (0–1)						0.79 (0.75–0.80)						0.82 (0.76–0.86)	0.052
	Depression Scale						7 (4-11)						6 (2.3–8)	0.346
Low-back pain-related														
	Duration of pain													
		<2 weeks	2.1 (1)						15 (3)					0.091
		2–12 weeks	34.0 (16)						25 (5)					
		>3–12 months	21.3 (10)						35 (7)					
		>12 months	42.6 20)						25 (5)					
	ODI (0–100)						24 (18–28)						21 (16.5–29)	0.611
Workability														
	Workability (0–10)						7 (6–8)						7.5 (6–8)	0.199
	Sick leave days ^d^		46.8 (22)						65 (13)					0.173
	Sick leave ^d^						7 (4.8–24.3)						15 (7–24.5)	0.088
	FABQ - work						12 (6–20)						15.5 (3–24)	0.597
	FABQ - physical activity						12 (9–14)						13 (9.8–15.8)	0.151

^a^ P-value for between-group difference from Chi square test. ^b^ P-value for between-group difference from independent-samples T test. ^c^ P-value for between-group difference from Mann-Whitney U test. ^d^ Number of low-back pain related sick leave days during preceding 3 months.

**Table 1d:** Baseline characteristics of participants in the trial arms stratified by high-risk work disability groups based on the short form of Örebro Musculoskeletal Pain Screening Questionnaire (ÖMPSQ-SF). **Bolded scores denote statistical significance with P<0.05**. [EQ=EuroQol score; FABQ=Fear Avoidance Beliefs Questionnaire; IQR=interquartile range; ODI=Oswestry Disability Index; SD=standard deviation].

			High-risk (N=80)											
			Intervention (N=58)						Control (N=22)					P-value
			% (N) ^a^		Mean (SD) ^b^		Median (IQR) ^c^		% (N) ^a^		Mean (SD) ^b^		Median (IQR) ^c^	
General health														
	Age (years)				46.6 (8.8)						44.7 (10.6)			0.171
	Female sex		58.6 (34)						68.2 (15)					0.433
	Body mass index (kg/m^2^)				28.9 (5.9)						28.2 (4.0)			0.080
	Smoking		22.7 (5)						21.1 (4)					0.575
	Self-rated health (1–100)						60 (50–70)						65 (56–70)	0.241
	EQ-5D (0–1)						0.70 (0.69–0.78)						0.70 (0.65–0.76)	0.325
	Depression Scale						10 (5–15)						9 (4.5–19)	0.953
Low-back pain-related														
	Duration of pain													
		<2 weeks	**0 (0)**						**4.5 (1)**					**0.014**
		2–12 weeks	**17.2 (10)**						**45.5 (10)**					
		>3–12 months	**25.9 (15)**						**22.7 (5)**					
		>12 months	**56.9 (33)**						**27.3 (6)**					
	ODI (0–100)						31 (25.5–44)						31 (27.5–40.5)	0.783
Workability														
	Workability (0–10)						6 (3–7)						6 (4–7.25)	0.458
	Sick leave days ^d^		62.1 (36)						72.7 (52)					0.372
	Sick leave ^d^						28 (7–75)						12 (5.5–24)	0.947
	FABQ - work						19 (10–26)						20.5 (10.8–28.3)	0.779
	FABQ - physical activity						14 (12–17.3)						17 (14–20)	0.114

^a^ P-value for between-group difference from Chi square test. ^b^ P-value for between-group difference from independent-samples T test. ^c^ P-value for between-group difference from Mann-Whitney U test. ^d^ Number of low-back pain related sick leave days during preceding 3 months.

### OHS visits, resource use, and sick leave days

During the one-year follow-up, the median number of visits to physicians was 1 (IQR 0–3) in the intervention arm and 2 (IQR 1–4) in the control arm, while the median number of physiotherapist visits was 2 (IQR 1–3) and 1 (IQR 0–2), respectively (table 2a). In the intervention arm, 90.1% (N=209) received multiprofessional physiotherapist-driven care compared to 63.7% (N=51) in the control arm (P<0.001; table 2b). In the high-, medium- and low-risk groups use of multiprofessional physiotherapist-driven care was higher in the intervention arm (89.7% versus 91.4% versus 89.7%, respectively) than the control arm (50% versus 65% versus 71.1%, respectively; table 2b).

**Table 2a:** Occupational health (OH) service visits and sick leaves in the trial arms during 1-year follow-up, stratified by short form of Örebro Musculoskeletal Pain Screening Questionnaire (ÖMPSQ-SF) risk groups for work disability. [IQR=interquartile range].

		Total				Low risk				Medium risk				High risk		
		Intervention (N=232)		Control (N=80)		Intervention (N=127)		Control (N=38)		Intervention (N=47)		Control (N=20)		Intervention (N=58)		Control (N=22)
		Median (IQR)		Median (IQR)		Median (IQR)		Median (IQR)		Median (IQR)		Median (IQR)		Median (IQR)		Median (IQR)
All physician visits		1 (0–3)		2 (1–4)		1 (0–2)		1 (1–3.3)		1 (0–3)		2 (1–3)		2 (1–5)		3 (1.8–5)
	Specialist physician in OH	1 (0–2)		1 (0–3)		1 (0–2)		1 (0–2)		1 (0–3)		1.5 (1–3)		2 (0.8–3)		1.0 (0–4)
	Physician without specialization in OH	0 (0–0)		0 (0–0)		0 (0–0)		0 (0–0)		0 (0–0)		0 (0–0)		0 (0–0)		0 (0–1)
	Remote physician visits	0 (0–1)		0 (0–1)		0 (0–0)		0 (0–0)		0 (0–1)		0 (0–1)		1 (0–1.3)		0 (0–1)
All OH physiotherapist visits		2 (1–3)		1 (0–2)		2 (1–3)		1 (0–2)		2 (2–3)		1 (0–2.8)		2 (1–4)		0.5 (0–3)
	Remote OH physiotherapist visits	0 (0–1)		0 (0–0)		0 (0–1)		0 (0–0)		0 (0–1)		0 (0–0)		0 (0–1)		0 (0–0)
OH nurse visits		0 (0–0)		0 (0–0)		0 (0–0)		0 (0–0)		0 (0–0)		0 (0–0)		0 (0–0)		0 (0–0)
Total OH service resource use		3 (2–5.8)		3 (2–5)		3 (2–5)		3 (1.8–5)		3 (2–6)		3.5 (2–5.8)		5 (3–8)		4.5 (2.8–7.3)
Number of sick leave days		0 (0–5)		1 (0–11)		0 (0–2)		0 (0–4.3)		0 (0–7)		1.5 (0–11.5)		2.5 (0–24.8)		7 (1.5–50.8)

**Table 2b:** Occupational health service resource use in the trial arms during 1-year follow-up, stratified by short form of Örebro Musculoskeletal Pain Screening Questionnaire (ÖMPSQ-SF) risk groups for work disability. [HCP=healthcare professional].

		Total					Low risk					Medium risk					High risk			
		Intervention (N=232)		Control (N=80)	P-value		Intervention (N=127)		Control (N=38)	P-value		Intervention (N=47)		Control (N=20)	P-value		Intervention (N=58)		Control (N=22)	P-value
		% (N) ^a^		% (N) ^a^			% (N) ^a^		% (N) ^a^			% (N) ^a^		% (N) ^a^			% (N) ^a^		% (N) ^a^	
Multiprofessional physio- therapist-driven care		90.1 (209)		63.7 (51)	<0.001		89.7 (114)		71.1 (27)	<0.001		91.4 (43)		65.0 (13)	0.003		89.7 (52)		50.0 (11)	<0.001
	Physiotherapist and physician or nurse visits	82.8 (196)		57.5 (46)	<0.001		85.8 (109)		65.8 (25)	0.006		85.1 (40)		55.0 (11)	0.008		81.0 (47)		45.5 (10)	0.002
	Physiotherapist and physician and nurse visits	5.6 (13)		6.3 (5)	0.858		3.9 (5)		5.3 (2)	0.744		6.4 (3)		10.0 (2)	0.599		8.6 (5)		4.5 (1)	0.513
Physician-driven care		6.9 (16)		33.8 (27)	<0.001		7.9 (10)		28.9 (11)	<0.001		4.3 (2)		30.0 (6)	0.003		6.9 (4)		45.5 (10)	<0.001
	Physician and nurse visits	0.4 (1)		6.3 (5)	0.001		0		7.8 (3)	0.001		2.1 (1)		5 (1)	0.527		0		4.5 (1)	0.102
	Physician visits only	6.5 (15)		27.5 (22)	<0.001		7.9 (10)		21.0 (8)	0.022		2.1 (1)		25.0 (5)	0.003		6.9 (4)		40.9 (9)	<0.001
Nurse visits only		0		1.3 (1)	0.088		0		0			0		0			0		4.5 (1)	0.102
No visits to HCP after recruitment		3.0 (7)		1.3 (1)	0.388		2.4 (3)		0	0.339		4.3 (2)		5.0 (1)	0.893		3.4 (2)		0	0.378
Total percentages (frequencies)		100 (232)		100 (80)			100 (127)		100 (38)			100 (47)		100 (20)			100 (58)		100 (22)	

^a^ P-value for between-group difference from Chi square test.

Both physician and physiotherapist were seen in total in 158 cases including 122 (52.6%) patients in the intervention arm, and 36 (45.0%) in the control arm, respectively. All three (physician, nurse and physiotherapist) were seen in 18 cases including 13 (5.6%) patients in the intervention arm and 5 (6.3%) of patients in the control arm, respectively. The number and percentage of patients having contact only with a physician was 15 (6.5%) in the intervention arm and 22 (27.5%) in the control arm, while 1 person (1.3%) had contact with a nurse in the control arm (P<0.001).

**Figure 2:**
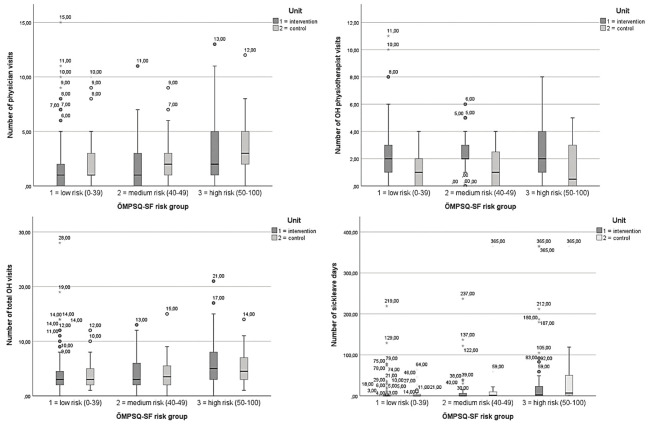
The occupational health services resource use and number of sick leave days in the trial arms, stratified by short form of Örebro Musculoskeletal Pain Screening Questionnaire (ÖMPSQ-SF risk groups). [OH=occupational health].

The median number of sick leave days in the intervention arm was 0 (IQR 0–5) and 1 (IQR 0–11) in the control arm. In the intervention arm, 64.7% of patients had no sick leave due to LBP over the 1-year follow-up period compared to 50% in the control arm. Figure 2 shows the use of OHS resources and sick leave days (minimum, maximum, median and percentiles) in the trial arms stratified by ÖMPSQ-SF risk groups.

### Costs

Table 3 presents the main results of the study in the trial arms for the total study sample and stratified for the ÖMPSQ-SF risk groups. The median cost of all OHS resource use was €222 (IQR €144–389) per patient in both the control and intervention arms. The intervention arm accrued significantly lower costs for physician visits (adjusted mean difference: €-43, 95% CI €-82–-3) but higher costs for physiotherapist visits (€55, €26–84) compared with the control arm. Total costs did not differ statistically significantly between the arms €-1908 (€-6734–2919). Supplementary table S2 presents a comparison of total costs between trial arms including the estimated training costs.

**Table 3:** Adjusted comparison of mean costs by trial arm for the total study sample and stratified by short form of Örebro Musculoskeletal Pain Screening Questionnaire (ÖMPSQ-SF) risk groups. The mean difference presents the intervention effect. Negative mean difference indicates lower costs and positive mean difference indicates higher costs for the intervention arm. [LBP=low back pain; X-ray=radiograph; MRI=magnetic resonance imaging; ENG=electroneurography; ENMG=electromyoneurography; OHS=occupational health service].

Patient group	Sources of costs (see supplement table 1)	Median (IQR) estimated cost (€)		Mean difference ^a^ (95% confidence interval)	P-value ^a^
		Intervention	Control		
Total (N=312)	Physician visits ^b^	66 (0–167)	101 (66–256)	-43 (-82–-3)	0.034
	Physiotherapist visits ^b^	148 (74–222)	74 (0–148)	55 (26–84)	<0.001
	Nurse visits ^b^	0 (0–0)	0 (0–0)	-2 (-7–3)	0.451
	Imaging due to LBP (X-ray/ MRI/ ENG/ENMG) ^c^	0 (0–0)	0 (0–0)	-5 (-27–18)	0.682
	OHS resource use ^b^	222 (148–392)	222 (134–372)	12 (-51–75)	0.714
	Sick leaves (€350/day) ^b^	0 (0–1750)	350 (0–3850)	-1916 (-6715–2883)	0.430
	Total costs ^b^	257 (148–2094)	807 (183–4172)	-1908 (-6734–2919)	0.435
Low risk (N=165)	Physician visits ^c^	66 (0–101)	66 (66–199)	-46 (-98 – 5)	0.075
	Physiotherapist visits ^c^	148 (74–222)	74 (0–148)	49 (11–88)	0.012
	Nurse visits ^c^	0 (0–0)	0 (0–0)	-1 (-7–6)	0.840
	Imaging due to LBP (X-ray/ MRI/ ENG/ENMG) ^c^	0 (0–0)	0 (0–0)	15 (-17–48)	0.354
	OHS resource use ^c^	214 (140–315)	214 (74–338)	23 (-63–109)	0.591
	Sick leaves (€350/day) ^c^	0 (0–700)	0 (0–1488)	580 (-2481–3640)	0.709
	Total costs ^c^	222 (148–840)	232 (130–1725)	616 (-2497–3728)	0.697
Medium risk (N=67)	Physician visits ^d^	66 (0–198)	132 (66–198)	-60 (-145- 24)	0.160
	Physiotherapist visits ^d^	148 (109–222)	74 (0–204)	52 (0–104)	0.049
	Nurse visits ^d^	0 (0–0)	0 (0–0)	-5 (-13–3)	0.254
	Imaging due to LBP (X-ray/ MRI/ ENG/ENMG) ^c^	0 (0–0)	0 (0–0)	-3 (-42–37)	0.877
	OHS resource use ^d^	222 (148–413)	257 (134–363)	-1 (-117–115)	0.990
	Sick leaves (€350/day) ^d^	0 (0–2450)	525 (0–4025)	-3100 (-13720–7520)	0.562
	Total costs ^d^	411 (148–413)	749 (227–4205)	-3088 (-13736–7560)	0.564
High risk (N=80)	Physician visits ^e^	132 (66–253)	198 (93–299)	-26 (-112–59)	0.541
	Physiotherapist visits ^e^	148 (74–257)	37 (0–222)	57 (-10–123)	0.094
	Nurse visits ^e^	0 (0–0)	0 (0–0)	-1 (-11–10)	0.883
	Imaging due to LBP (X-ray/ MRI/ ENG/ENMG) ^c^	0 (0–0)	0 (0–27)	-43 (-90–4)	0.072
	OHS resource use ^e^	284 (179–558)	315 (186–622)	7 (-131–145)	0.921
	Sick leaves (€350/day) ^e^	875 (0–8663)	2450 (525–17763)	-1413 (-15119–12292)	0.838
	Total costs ^e^	1196 (199–9096)	2801 (942–18220)	-1406 (-15165–12352)	0.839

^a^ Bootstrapped (2500 replications). ^b^ Linear mixed model with covariate adjustment for pain duration and FABQ-Physical activity. ^c^ Linear mixed model with adjustment for age. ^d^ Linear mixed model with adjustment for general health. ^e^ Linear mixed model with adjustment for pain duration.

In the low- and medium-risk groups, physiotherapist costs were higher in the intervention than control arm (adjusted mean difference €49, 95% CI €11–88 in the low-risk group, and €52, 95% CI €0–104 in the medium-risk group). Sickness absence costs seemed to be lower in the intervention arm among patients with medium- and high-risk of work disability but not statistically significantly compared to the control arm. Supplementary table S3 presents the unadjusted associations between trial arms and estimated costs.

The analysis comparing visits, sick leave days, total estimated OHS resource use and costs between the ÖMPSQ-SF risk groups in the total sample combining intervention and control arms is shown in supplementary table S4. The median number of OHS visits and days on sick leave differed statistically significantly between the groups. For example, the median number of days on sick leave were higher in the high- and medium-risk ÖMPSQ-SF groups [5 (IQR 0–34) and 0 (IQR 0–7) days, respectively] than in the low-risk group [0 (IQR 0–3) days].

There were also statistically significant differences in the OHS resource utilization and sick leave costs between the ÖMPSQ-SF risk groups. For instance, compared to the low-risk group, the total costs for patients in the high-risk group were more than five-fold higher with intervention arm [€1196 (IQR €199-9096); versus €222 (IQR €148-840)] and as much as twelve-fold higher in the control arm [€2801 (IQR €942-18220); versus €232 (130-1725), respectively].

## Discussion

The current study was, to our knowledge, the first to evaluate subsequent changes in OHS resource use and costs after implementing a guideline-based BPS model for managing patients with LBP in the OH primary care setting. The study suggests that a brief BPS training intervention for OH professionals may help to shift OHS resources towards multiprofessional physiotherapist-driven care, instead of solely physician-driven care, without significant differences in total costs. Stratification according to ÖMPSQ-SF risk groups showed that in the low- and medium-risk groups of work disability, physiotherapist costs were higher in the intervention than control arm, but no statistically significant differences were observed between the arms either in the average total resource utilization or the sickness absence costs.

Higher use of physiotherapist resources in the LBP management might imply that patients receive more concrete guidance on self-care and exercises for functional restoration, advice to cope with pain, and encouragement for staying active and returning to work. For patients with LBP, the approximate length of one visit with an OH physician is 15–20 minutes versus 60 minutes with an OH physiotherapist (supplementary table S1). This may provide an added opportunity for learning and getting support also on the BPS aspects of their conditions. In terms of OHS resource use, a shift towards more physiotherapist-driven care for patients with LBP could increase the availability of physician resources for other prioritized patient groups in OHS.

There is a paucity of research on the resource use in the OHS among patients with LBP, and due to the diversity of national systems, the generalizability and applicability of results is limited internationally. A recent Finnish study (N=87 468) examined the use of OHS by diagnosis category showing that patients diagnosed with musculoskeletal disorders (MSD) or/and mental disorders (MD) differed from other workers in their use of OHS and cost of sick leaves. The most common MSD diagnosis was LBP (M40–54), diagnosed in 71% of patients (N=9557) where approximately half also had MD. Up to 30% of patients with MSD had not used the services of an OH physiotherapist at all during the 3-year follow-up period. Employees diagnosed with both MSD and MD accounted for 73% of costs (21). The average OHS cost per patient with LBP in 2017 was nearly two-fold higher with the combination of LBP and MD (Dr. M. Lahelma, personal communication 13.12.2023). In our study, the total costs were five-fold higher with BPS care (€1196 versus €222) and as much as twelve-fold higher under conventional care (€2801 versus €232) for the patients with high risk of work disability compared to those with low risk. Given that the ÖMPSQ-SF pays attention to BPS factors, the patients with high risk of work disability in our cohort might have similarly complex and manifold needs to the population diagnosed with MSD and MD in the abovementioned study. The high-risk patients clearly represent a group with need of a broad multiprofessional care to avoid disability.

Extending working careers has become an important objective in aging societies (22), and, thus, identifying patients with an increased risk of work disability at early stage and providing targeted care is crucial. A systematic review demonstrated that a stratified care approach based on STarT Back Tool for patients with LBP provides substantial clinical and health-related cost benefits in the medium- and high-risk subgroups compared with physiotherapy without stratification in primary care (23, 24). Our study supports the findings concerning stratification and encourages the use of ÖMPSQ-SF in the OHS setting to aid decision-making concerning rational allocation of healthcare resources for different subgroups of LBP patients.

Furthermore, our approach combines both work disability risk stratification and BPS management. In the Finnish context, even the Finnish Ministry for Social Affairs and Health recently recommended that BPS rehabilitation for prolonged or recurrent LBP should be routinely offered in the publicly funded health services (25). The recommendation does not specify the roles of the different HCP, but our study suggests that a BPS model can be implemented in the form of multiprofessional physiotherapist-driven care in both public and private OHS (26). Previous studies from the primary care settings have also reported that having a physiotherapist as the primary assessor for patients with MSD appears to be as cost-effective as physician-driven usual care (27–29).

The role of an OHS in LBP management is to assess the patient’s workability in relation to their work circumstances and ensure that the necessary support and rehabilitation measures are taken. In Finland, workability support for LBP patients includes services provided by the OH physician (eg, consultations, medical examination and treatment, workability assessment, prescriptions of partial and full sick leave), rehabilitative support provided by OH physiotherapist (eg, counselling and advice on self-care methods for pain management, functional restoration interventions), and workplace activities by both professions as agreed upon and planned with the workplace (eg, OH collaborative negotiations, workplace assessments and recommendations of work modifications) (30, 31). The widest possible use of different forms of workability support should be directed at patients with elevated risk of work disability, yet 6.9% of high-risk patients in the intervention arm and 45.5% in the control arm received physician-driven single-professional care (P=<0.001). Instead of the previous physician-only or physician-to-physiotherapist practice model, a physiotherapist-only, physiotherapist-to-physician – in case of ‘red flags’ or a more challenging pain problem – or a more multiprofessional approach, including also a nurse, psychologist or social worker according to the needs, could be implemented in OHS.

The strength of our study is that the cost analysis is conducted on the basis of a cluster randomized controlled trial. This design also offers an opportunity to evaluate the OHS resource use and costs under real world conditions, with recruited patients representative of typical clinical caseloads, a comparison of the intervention with current OHS practice, and follow-up under routine conditions (32). The study involved key nationwide OHS organizations with 27 separate OHS units. Objective data from individual EPR provided complete information on the OHS resource use, based on the mandatory registration of visits and sick leaves for a specific patient group of public health relevance. The limited number of exclusion criteria enhances the generalizability of the results from an OHS setting to a wider primary healthcare context. However, our study population presented with relatively mild risk pattern for work disability, which warrants consideration in terms of generalizability.

Our study has some limitations as well. Patients in the intervention and control arms differed at baseline in terms of age, general health, and pain duration, especially in the ÖMPSQ-SF risk groups. However, we attempted to control for these observed imbalances in the primary analyses. Although the approximate total number of clients served by the units was slightly over 150 000 in both trial arms, substantially less participants were recruited in the control versus intervention arm. In the control arm, approximately 1 per 1250 clients served gave consent to participate, compared to 1 per 450 in the intervention arm, which indicates recruitment was likely affected by selection bias (33, 34). Moreover, 34% of intervention arm patients' pain lasted >12 months compared to 17% in the control arm (15). A critical factor affecting the completion of the study was the slow pace of patient recruitment. This occurred despite our effort to design the inclusion and exclusion criteria as pragmatic and simple as possible, aiming to minimize the workload for professionals within the participating OHS units. The training intervention was free of direct costs to the OHS providers and participants. It is easy to think of the intervention as a form of voluntary continuous education typically provided by OH employers to their employees, which could also be considered as an investment of time for better care, which is why we did not include training costs in the main analysis. The training costs can be seen as a one-time investment for better division of work and well-being at work. Nevertheless, training costs are a relevant issue for wider implementation, and we also undertook an additional sensitivity analysis by adding an artificially high cost per patient to cover the estimated costs of training (supplementary table S2). The cost of training on a per-patient basis would likely be much lower, as training would also be likely to contribute to the care many future patients. Even when using an upper-end estimate of €126 per patient, our base-case findings were not altered to any substantial extent.

In this study, we did not have access to records of OHS resource use or sickness absence for diagnoses other than LBP. We could not evaluate either the role of presenteeism or the KELA reimbursements provided to employers. We assume that these factors impacted both study arms equally. If any impacts were present, they likely contributed to cost reductions for employers. Additionally, the use of partial sick leave likely reduced costs, but this was not common in our data, with partial sick leave being prescribed for only 3.8% (N=9) and 2.5% (N=2) of patients in the intervention and control groups, respectively. It is important to note that the association of LBP and MD and their combination is linked to more frequent use of healthcare resources (21, 35) and work disability (3, 36, 37). Even though trial-based analyses often have limitations, the study provided tools for professionals to identify BPS risk factors for disability and to prioritize the use of OHS resources optimally based on individual needs. Partly due to regulatory problems outside our control and incertitude caused by selection biases in this cluster-randomized trial, we were not able to obtain sufficient data to conduct a full economic evaluation in a sufficiently robust manner. Therefore, we combined estimated healthcare-payer and sick-leave costs to form a cost analysis from a limited societal perspective, thus providing useful new evidence comparing some of the costs associated with usual care to those of a guideline-oriented BPS approach to management of LBP.

The evaluation and treatment process of guideline-based BPS management (supplementary figure S1) took into account the multidimensional nature of LBP (38). The percentage of trained physiotherapists was 44% but the respective coverage for physicians was only 2% (analysis not shown). This is a limitation for implementation of the BPS model in the intervention units. However, the training participants were urged to use and champion the BPS model to guide and support colleagues. The BPS model of care may be more easily implemented in the future since the intervention has now been tested in a real clinical OHS setting (39, 40).

Importantly, our previous study serving as the platform of the present study was designed to enhance a new approach to pain management in the OH primary care setting. In the future, an agile subgrouping of patients could be useful in the implementation of patient-centered treatment pathways. Reorganizing LBP management in OH primary care could help to manage physician workload and promote a more efficient use of OHS resources. As the high-risk patients might have multiple risk factors for work disability, their OHS care should be carefully planned and coordinated, including the continuity of multiprofessional BPS care and return-to-work coordination.

### Concluding remarks

With no substantial difference in total costs, brief BPS training may help LBP patients with differing risks of work disability by shifting OHS resources from solely physician- to multiprofessional physiotherapist-driven care. Implementation research is needed to further analyze the use of stratification by work disability risk, with pre-defined criteria for defining adherence to the intended model of LBP care.

## Supplementary material

Supplementary material
