# Supplementary material for: Cost analysis comparing guideline-oriented biopsychosocial management to usual care for low-back pain: a cluster-randomized trial in occupational health primary care
Source: Scand J Work Environ Health. 2025 Apr 27;51(3):201–13. doi: 10.5271/sjweh.4212 (PMC12070492; doi:10.5271/sjweh.4212)
Supplement: Supplementary material [file SJWEH-51-201-S001.pdf]

Cost analysis comparing guideline-oriented biopsychosocial management to usual care for low-back pain: a cluster-randomized trial in occupational health primary care1

by Maija Paukkunen, MSc,[1, 2] Jaro Karppinen, MD, PhD,[2, 3] Birgitta Öberg, PhD,[1] Leena Ala-Mursula, MD, PhD,[4] Eveliina Heikkala, MD, PhD,[4, 5] Katja Ryynänen, MD, PhD,[4] Riikka Holopainen, PhD,[6, 7] Samuel Booth,[4] Neill Booth, PhD,[8] Allan Abbott, PhD [1,9]

1. Supplementary material
2. Research Unit of Health Sciences and Technology, PO Box 8000, University of Oulu, 90014, Oulu, Finland. [E-mail: maija.paukkunen@oulu.fi]

Table S1. Occupational healthcare (OH) unit costs in 2017.

Resource	Time (minutes)	Cost (€)
Physician visit in OH primary care [a]	15-20	66
Remote OH physician visit [a]	15-20	35
OH physiotherapist visit [a]	45-60	74
Remote OH physiotherapist visit [a]	15-30	35
OH nurse visit [a]	15-20	32
Magnetic resonance imaging (MRI) [a]		337
Radiograph (X-ray) [a]		106
Electroneuromyography (ENMG) [a]		106
Electroneurography (ENG) [a]		106
Cost estimate of sick leave day [b]		350

[a] Unit cost in 2017 (18)
[b] Estimated by the Confederation of Finnish Industries in 2017

Table S2. Additional sensitivity analysis with estimated training costs included.

Patient group	Sources of costs (see supplement table 1)	Median (IQR) estimated cost (€)		Mean difference [a] (95% confidence interval)	*P*-value [a]
		Intervention [c]	Control		
Total n=312	OHS resource use [b]	348 (274-518)	222 (134-372)	138 (246 - 350)	<0.001
	Sick leaves (€350/day) [b]	126 (126-1876)	350 (0-3850)	-1790 (-9189 - 2967)	0.591
	Total costs [b]	383 (274-2221)	807 (183-4172)	-1782 (-9498 - 3128)	0.590

OHS = Occupational health service
[a] bootstrapped (2500 replications)
[b] linear mixed model with covariate adjustment for pain duration and FABQ-Physical activity
[c] a one-time investment of €29222 of providing the training is included in the analysis (€126 per patient recruited from the intervention arm).

The costs of receiving the training were calculated according following estimates: Ten OH physicians spent average of 24.6 hours in the training costing €51/hour. Twenty-two OH physiotherapists spent average of 33.3 hours in the training costing €18/hour. The median monthly salary of OH physicians in 2017 was €7800, as estimated by the Finnish Medical Association. The median monthly salary of OH physiotherapists in 2017 was €2800, as estimated by the Finnish Association of Occupational Health Physiotherapists. The cost of participating to trainings were €25722. Adding this to the costs of training (€3500) is a one-time investment of €29222, equivalent to €126 per patient recruited in the intervention arm.

Table S3. Unadjusted comparison of median costs and mean differences in trial arms, stratified by short form of Örebro Musculoskeletal Pain Screening Questionnaire (ÖMPSQ-SF) risk groups. The mean difference presents the intervention effect. Negative mean difference points to less costs for intervention arm, positive mean difference for more costs.

Patient group	Cost estimate [b]	Median (IQR) cost (€)		Mean difference (95% confidence interval) [a]	*p*-value [a]
		Intervention	Control		
All n=312	Physician visits	66 (0-167)	101 (66-256)	-43 (-82 - -3)	**0.034**
	Physiotherapist visits	148 (74-222)	74 (0-148)	55 (26—84)	**<0.001**
	Nurse visits	0 (0-0)	0 (0-0)	-2 (-7 – 3)	0.451
	Imaging due to LBP	0 (0-0)	0 (0-0)	-5 (-27 - 18)	0.682
	OHS resource use	222 (148-392)	222 (134-372)	12 (-51 – 75)	0.714
	Days of sick leave	0 (0-1750)	350 (0-3850)	-1916 (-6715 – 2883)	0.430
	Total costs	257 (148-2094)	807 (183-4172)	-1908 (-6734 – 2919)	0.435
Low risk n=165	Physician visits	66 (0-101)	66 (66-199)	-46 (-98 – 5)	0.075
	Physiotherapist visits	148 (74-222)	74 (0-148)	49 (11 - 88)	**0.012**
	Nurse visits	0 (0-0)	0 (0-0)	-1 (-7 - 6)	0.840
	Imaging due to LBP	0 (0-0)	0 (0-0)	15 (-17 - 48)	0.354
	OHS resource use	214 (140-315)	214 (74-338)	23 (-63 - 109)	0.591
	Days of sick leave	0 (0-700)	0 (0-1488)	580 (-2481 - 3640)	0.709
	Total costs	222 (148-840)	232 (130-1725)	616 (-2497 - 3728)	0.697
Medium risk n=67	Physician visits	66 (0-198)	132 (66-198)	-60 (-145- 24)	0.160
	Physiotherapist visits	148 (109-222)	74 (0-204)	52 (0 - 104)	**0.049**
	Nurse visits	0 (0-0)	0 (0-0)	-5 (-13 - 3)	0.254
	Imaging due to LBP	0 (0-0)	0 (0-0)	-3 (-42 - 37)	0.877
	OHS resource use	222 (148-413)	257 (134-363)	-1 (-117 - 115)	0.990
	Days of sick leave	0 (0-2450)	525 (0-4025)	-3100 (-13720 - 7520)	0.562
	Total costs	411 (148-413)	749 (227-4205)	-3088 (-13736 - 7560)	0.564
High risk n=80	Physician visits	132 (66-253)	198 (93-299)	-26 (-112 - 59)	0.541
	Physiotherapist visits	148 (74-257)	37 (0-222)	57 (-10 - 123)	0.094
	Nurse visits	0 (0-0)	0 (0-0)	-1 (-11 - 10)	0.883
	Imaging due to LBP	0 (0-0)	0 (0-27)	-43 (-90 - 4)	0.072
	OHS resource use	284 (179-558)	315 (186-622)	7 (-131 - 145)	0.921
	Days of sick leave	875 (0-8663)	2450 (525-17763)	-1413 (-15119 - 12292)	0.838
	Total costs	1196 (199-9096)	2801 (942-18220)	-1406 (-15165 - 12352)	0.839

ÖMPSQ-SF = Short form of Örebro Musculoskeletal Pain Screening Questionnaire; IQR = interquartile range; LBP = low back pain

[a] Bootstrapped 2500 replications

[b] Linear mixed models, unadjusted

Table S4. Differences in visits, sick leave days and costs between risk groups based on the short form Örebro Musculoskeletal Pain Screening Questionnaire (ÖMPSQ-SF) scores in the whole study sample.

	Low risk n = 165	Medium risk n = 67	High risk n = 80	*P*-value [b]
Number of visits and sick leave days				
OHS visits [a]	3 (2–5)	3 (2–6)	5 (3–8)	**<0.001**
Sick leave days [a]	0 (0–3)	0 (0–7)	5 (0–34)	**<0.001**
Costs				
Total costs of OHS resource use [a]	249 (148-478)	296 (175-764)	746 (253-1406)	**<0.001**
Sick leave costs [a]	0 (0–150)	0 (0–650)	250 (0–1825)	**<0.001**

OHS = Occupational health services
[a] Median (interquartile range)
[b] Kruskall-Wallis H test

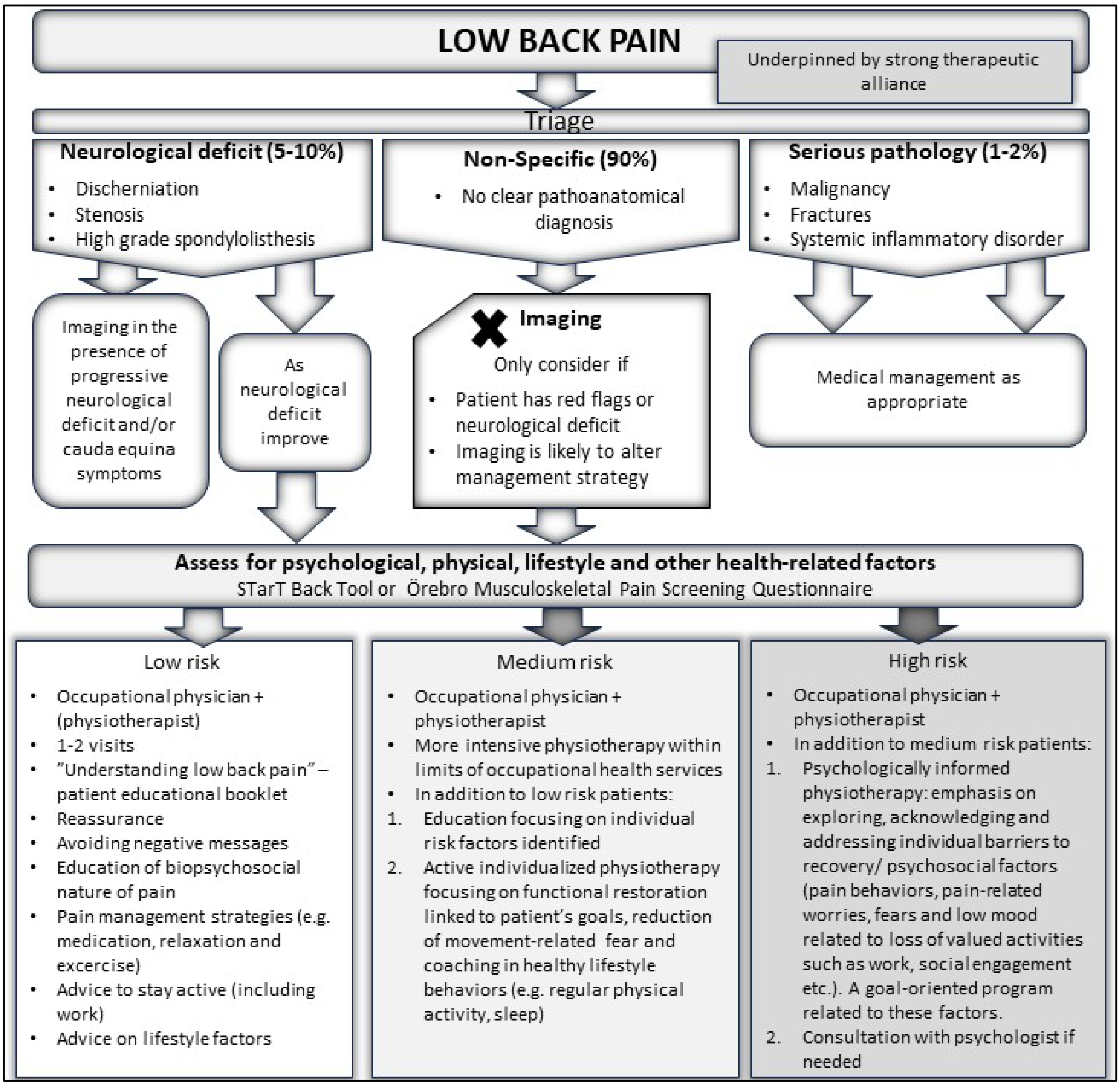

Figure S1. Infographic of evaluation and treatment process of guideline-based biopsychosocial management of low back pain.

Örebro Musculoskeletal Pain Screening Questionnaire (Short-form)

Name____ Date____

1. How long have you had your current pain problem? Tick (√) one.

0-1weeks[1] □	1-2weeks[2] □	3-4weeks[3] □	4-5weeks[4] □	6-8weeks[5] □
9-11weeks[6] □	3-6months[7] □	6-9months[8] □	9-12months[9] □	over1year[10] □

2. How would you rate the pain that you have had during the past week? Circle one.

0	1	2	3	4	5	6	7	8	9	10
No pain									*Pain as bad as it could be*	

Please circle the one number which best describes your current ability to participate in each of these activities.

3. I can do light work for an hour.

0	1	2	3	4	5	6	7	8	9	10
Can't do it because of the pain problem									*Can do it without pain being a problem*	

4. I can sleep at night.

0	1	2	3	4	5	6	7	8	9	10
Can't do it because of the pain problem									*Can do it without pain being a problem*	

5. How tense or anxious have you felt in the past week? Circle one.

0	1	2	3	4	5	6	7	8	9	10
Absolutely calm and relaxed									*As tense and anxious as I've ever felt*	

6. How much have you been bothered by feeling depressed in the past week? Circle one.

0	1	2	3	4	5	6	7	8	9	10
Not at all										*Extremely*

7. In your view, how large is the risk that your current pain may become persistent?

0	1	2	3	4	5	6	7	8	9	10
No risk										*Very large risk*

8. In your estimation, what are the chances you will be working your normal duties in 3 months

0	1	2	3	4	5	6	7	8	9	10
No chance										*Very Large Chance*

Here are some of the things which other people have told us about their pain. For each statement, please circle one number from 0-10 to say how much physical activities, such as bending, lifting, walking, or driving affect your pain.

9. An increase in pain is an indication that I should stop what I'm doing until the pain decreases.

0	1	2	3	4	5	6	7	8	9	10
Completely disagree										*Completely agree*

10. I should not do my normal work with my present pain.

0	1	2	3	4	5	6	7	8	9	10
Completely disagree										*Completely agree*

Figure S2. Örebro Musculoskeletal Pain Screening Questionnaire (Short-form).
